# Experiment and Validation of Local Bearing Capacity for Ultra-High-Performance Concrete Confined with Stirrups

**DOI:** 10.3390/ma15175869

**Published:** 2022-08-25

**Authors:** Moneef Mohamed Elobaid Musa, Xueyu Xiong, Yang Zhang

**Affiliations:** 1 Department of Structural Engineering, Tongji University, Shanghai 200092, China; 2 Key Laboratory of Advanced Civil Engineering Materials of Ministry of Education, Tongji University, Shanghai 200092, China

**Keywords:** ultra-high-performance concrete, finite element model, bearing capacity, local area aspect ratio, reinforcement ratio

## Abstract

Ultra-high-performance concrete (UHPC) has the advantages of high compressive and tensile strength, high bending strength, good durability, remarkable corrosion resistance, and low self-weight. In this study, ten UHPC specimens were designed based on three fundamental parameters, including the ratio of the gross supporting area *A*_b_ to the bearing plate area *A*_l_ (local area aspect ratio *A*_b_/*A*_l_)_,_ the ratio of core area *A*_cor_ to the bearing plate area *A*_l_ (core area aspect ratio *A*_cor_/*A*_l,_)_,_ and the reinforcement ratio *p*_v_, to investigate mechanical behaviors and bearing capacity. Failure modes, cracking load, crack propagation, wedge features, the relationship between local compression and deformation, and the local bearing capacity was investigated. Finite element models (FEMs) were built to simulate and validate the observed behavior of the anchorage zone under compressive loading. The experiment results demonstrate that the *p*_v_ significantly increases the bearing capacity. When the reinforcement ratio increased from 4.5% to 3.7%, the bearing capacity increased by 23%, and the effect of *A*_cor_/*A*_l_ was not obvious. In addition, decreasing the *A*_b_/*A*_l_ from 11.1 to 6.3 increases the bearing capacity to 19%. Furthermore, a model was proposed to predict the bearing capacity of the UHPC specimens reinforced with stirrups. The calculated values, numerical predictions, and experiment results showed good agreement.

## 1. Introduction

Concrete is a major widely used material in structures. Various materials are used to improve the properties of concrete by enhancing industrial wastes [1], non-biodegradable granite Pulver [2], and modern methods by steel fiber such in engineered cementitious composite (ECC), steel fiber-reinforced concrete (SFRC), and ultra-high-performance concrete (UHPC). UHPC is a new type of cement-based composite material. It has recently become a research hotspot; Ed Larrard introduced it for the first time in 1994 [3]. UHPC mix contains less water ratio, steel fibers, a large amount of superplasticizer, high cementite component, and quartz sand [4]. The mechanical properties of UHPC can be enhanced by using mineral admixtures and ceramic wastes [5], and silica fume [6]. UHPC is suitable for significant marine projects, including military structures, long-span bridges [7], and nuclear power plants [8] due to its superior mechanical and durability characteristics [9]. UHPC composites generally offer high tensile and compressive strength of over 150 MPa [10]. Researchers have studied the mechanical properties of UHPC in the structural members, such as in the columns [11,12], beam [13], and slabs [14,15]. The application of UHPC in the prestressed structure can achieve lightweight members and increase the structural span [16,17].

In prestressed structures, the anchorage zone is exhibited to concentrated load due to the prestressing force transferred from steel strands to concrete through bearing plates and distributed across the whole member section; however, due to the existence of prestressing, the problem of local compression in the end anchorage zone of small-sized structures is more prominent.

The characteristics of prestressed concrete anchorage zones has been investigated by Burgoyne et al. [18]. An experimental study was conducted to determine the properties of concrete prisms loaded through rigid steel plates. This loading arrangement represents the force transfer of prestressed anchorages from tendons to concrete in concrete structures. Researchers have conducted comparative and systematic research on the local compression properties of ordinary concrete under the action of prestressing, such as bearing capacity [19], failure mechanism [20], and reinforcement design [21] in the anchorage zone. Ahmed et al. [22] introduced an equation that considers the size effect and reinforcement ratio to predict the local bearing capacity of reinforced concrete specimens. Haroon et al. [23] studied the use of steel fiber-reinforced concrete to reduce the secondary reinforcement in the anchorage zone. It showed that 1% content of 30 mm long hooked end steel fiber could replace 100% indirect reinforcement in the local anchorage area. Niyogi et al. investigated the influence of various parameters such as the reinforcement ratio [24], the bearing plate’s geometry and nature [25], and size [26]. The hoop strengthening theory, the wedge split theory, the tension–compression rod truss model, and other methods to describe concrete’s local compression failure mechanism and to calculate the local compression bearing capacity were put forward. Kim et al. verified the load transfer in the prestressed concrete anchorage area experimentally [27]. In order to provide the remarkable characteristics of UHPC compared with ordinary concrete, the cross-sectional size of the member was reduced, and the stress distribution, deformation and cracking mode of the post-tensioned anchorage zone were experimentally studied according to the reinforcement ratio of the member. Zhang et al. [28] tasted 39 RPC specimens to study the effects of the local compression area ratio on the local compression performance of RPC. The improvement coefficient of the RPC local compression strength was obtained. However, the test results have certain limitations due to the low strength of RPC and the relatively large local compression area. Zheng et al. [29] investigated the local compressive behavior of 24 steel fiber-reinforced RPC and 24 plain RPC specimens. The model for calculating the local bearing capacity was proposed considering the effect of the local area aspect ratio. Zhou et al. [30] found that the duct can significantly reduce RPC’s local compressive bearing capacity. Meanwhile, specimens reinforced with high-strength steel spirals [31] can improve the restraint effect on the core of the RPC, causing ductility failure. The calculation formula of the local compressive bearing capacity under different conditions was proposed.

Predicting the concrete’s local bearing capacity and failure mechanisms is necessary to optimize the local bearing capacity and provide an efficient design of anchorage zones in post-tensioned concrete members. The confinement effect provided by the surrounding concrete and reinforcements increases the compression strength of concrete at the local bearing capacity and resists bursting stress. In earlier studies, although a lot of research has been conducted to investigate the behavior of concrete confined with spirals under axial compression, studies on the effect of stirrups in concrete in anchorage zones are relatively limited, especially for stirrups in UHPC. Due to the short development time of UHPC and few prestressed UHPC structures, the research on the local compressive bearing capacity, failure characteristics, and crack development of UHPC is relatively lacking. Moreover, UHPC does not contain coarse aggregate but steel fiber. UHPC local compression failure mode and the calculation formula of the local compression bearing capacity should be significantly different from ordinary concrete. Therefore, it is important to investigate the effect of stirrups on the bearing capacity, develop a model to predict the bearing capacity of UHPC specimens confined with stirrups, and to build a finite element model to study the failure characteristics of UHPC specimens confined with stirrups.

In this article, ten UHPC specimens have been considered, and a local compression experiment was conducted to investigate the behavior of UHPC specimens reinforced with stirrups, the specimen’s failure mechanisms, crack patterns, and wedge features. The characteristics of the load–deformation curves were determined to assess the performance of the UHPC specimens in each loading stage. In addition, a finite element model was built to investigate the failure mechanism of UHPC specimens under local compression. Moreover, a model was proposed to predict the local bearing capacity of UHPC specimens based on the experiment results considering the effect of the stress generated by the stirrups. The accuracy of the proposed model and Finite element model (FEM) were validated using experimental data.

## 2. Experimental Program

### 2.1. Mechanical Properties of UHPC

**Figure 1:**
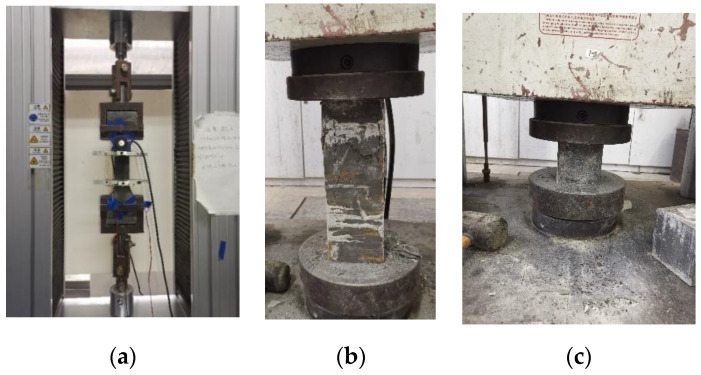
Tests for UHPC mechanical properties. (**a**) Dog bone specimen for tension test. (**b**) Prismatic specimen for compression test. (**c**) Cubic specimen for compression test.

The UHPC materials used in the test were composed of: (i) cement (P.II52.5), (ii) silica fume (SiO_2_ content is greater than 92%), (iii) quartz sand, (iv) fly ash (Class I), (v) mineral powder (S95), (vi) water reducer, and (iv) steel fiber. The steel fiber was 2.5% contained, with a diameter of 0.2 mm, and a length of 13 mm. The detailed proportions of the used UHPC are shown in Table 1. The mixing process of these materials was as follows: (a) the powder was added to the mixer bin in a single tray; (b) admixture and water were added according to the ratio of the single tray, and it was stirred for 4–5 min; (c) after the UHPC mixture appeared fluidized, the steel fiber was added slowly (steel fiber wasn’t added at once to avoid uneven dispersion), then continued to stir for 1~2 min after all fibers were added; and (d) due to the agglomeration of steel fiber, stirring was prolonged for 1~2 min until the steel fibers were evenly distributed, then the UHPC was poured. Following this, all the specimens had a 28-day natural cure. To determine the UHPC strength, 100 mm^3^ cubes, 100 mm × 100 mm × 300 mm prisms, and dog bone were tested. The mechanical properties of UHPC are as follows: the compressive strength for the cube was 166 MPa (*f*_cu_), and the prismatic compression strength (*f*_c_) was 140 MPa. Moreover, the tensile strength (*f*_t_) was 10 MPa, and the elastic modulus (*E*_s_) was 58,200 MPa. Figure 1 shows the dog bone, prismatic, and cubic specimens for the tensile and compression tests, respectively.

### 2.2. Mechanical Properties of Steel Bars

**Figure 2:**
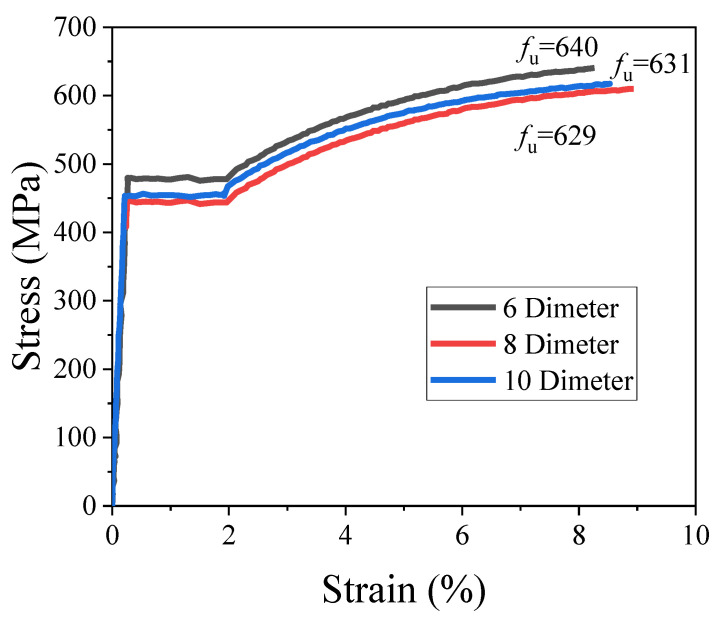
Steel bars stress–strain curve.

Grade 400 deformed bars with diameters of 6 mm, 8 mm, and 10 mm were utilized as longitudinal reinforcement for binding and as stirrups to confine the inner concrete. Table 2 and Figure 2 show the steel bar’s mechanical properties and the stress–strain curves.

### 2.3. Experimental Specimens Design

**Figure 3:**
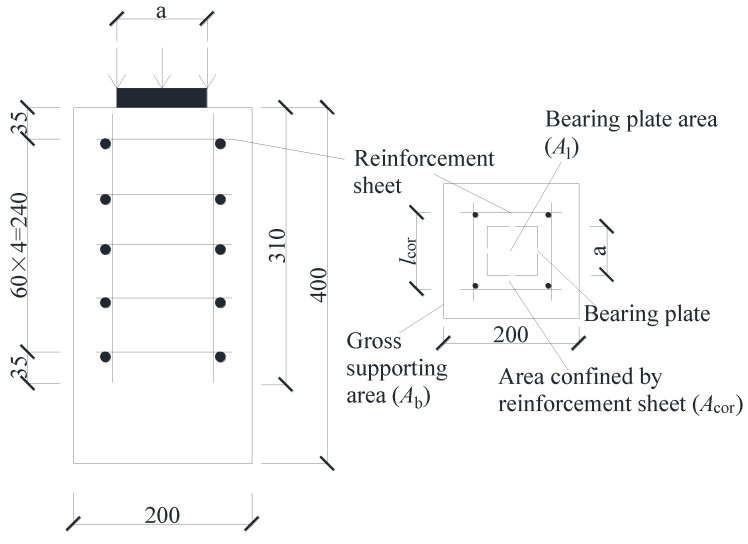
Schematic diagram of specimens (unit: mm).

According to Saint-Venant’s principle, the longitudinal compressive stress is uniformly distributed when the concentrated pressure diffusion distance equals the long side of the member’s section. The specimen was a prismatic UHPC block with a depth of 200 mm, a width of 200 mm, and a height of 400 mm, as shown in Figure 3. The specimen’s parameters are shown in Table 3, where *a* is the bearing plate length, *d* is the steel bar diameter, and *l*_cor_ is the length of the inner edge of the reinforcement sheet steel bar.

**Figure 4:**
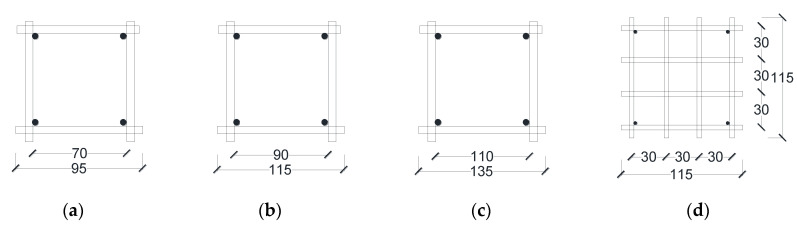
Reinforcement geometric dimensions. (**a**) *l*_cor_ = 70 mm; (**b**) *l*_cor_ = 90 mm; (**c**) *l*_cor_ = 110 mm; (**d**) *l*_cor_ = 90 mm.

Considering the influence of different length-bearing plates on the local compression of UHPC can be established. The minimum bearing plate size required by UHPC to achieve the local compressive bearing capacity of the section was obtained to meet the construction requirements on the premise of satisfying the design section. The three bearing plates have various dimensions: 60 mm, 70 mm, and 80 mm, respectively. The levels 11.1, 8.2, and 6.3 were used for the area aspect ratio *A*_b_/*A*_l_, *A*_cor_/*A*_l_ is the ratio that affects the local compression failure mode, where *A*_cor_ (*A*_cor_ = *l*_cor_*^2^*) is the core concrete area within the inner surface of the reinforcement, and *A*_l_ (*A*_l_ = *a^2^*) is the local area aspect. The length of the inner edge of the reinforcement sheet steel bar *l*_cor_ was as follows: 70 mm, 90 mm and 110 mm. As shown in Figure 4, the steel bar diameters were 6 mm, 8 mm and 10 mm.

**Figure 5:**
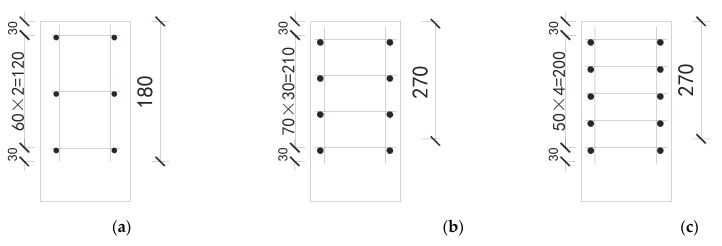
Reinforcement sheets arrangement. (**a**) 3 stirrups; (**b**) 4 stirrups, reinforcement mesh; (**c**) 3 reinforced mesh.

The research shows that when the same number of steel sheets are evenly arranged near the half-height range of the bearing plate, the local compression bearing capacity increases the most. Therefore, 300 mm downward from the local compression loading end of the specimen, various reinforcement sheets were arranged around as follows: 3 stirrups, 4 stirrups, 4 reinforcement mesh, and 5 stirrups, respectively. The distance between the first row and the end was 30 mm, and the distance between adjacent reinforcement was 50 mm, 60 mm, and 70 mm, respectively. The reinforcement was designed and fabricated of four Grade 400 deformed bars with a diameter of 6 mm, 8 mm, and 10 mm, which were used as stirrups. There were 4 Grade 400 deformed steel bars with a diameter of 6 mm, 8 mm, and 10 mm, which were used as erecting bars between each reinforcement sheet and were tied with the reinforcement sheets to form a steel skeleton. The reinforcement sheets arranged in the specimens are shown in Figure 5.

The reinforcement ratio of sheets can be calculated by *p*_v_ = *nA*_s_/(*l*_cor_*s*), the corresponding *p*_v_ reinforcement ratio is 0% to 6.4%, where *n* is the number of steel bars of the reinforcement, *A*_s_ is the cross-sectional area of a single steel bar in the reinforcement sheets, and *s* is the sheet spacing.

### 2.4. Experimental Setup and Loading Protocol

The estimated maximum failure load in the test was 2000 kN, so the YAW-5000J hydraulic pressure testing machine was selected, which can be loaded to 3000 kN. There is a spherical hinge on the top plate of the press, which is convenient for the centering of the test piece. A load-displacement control method was implemented in accordance with GB 50,152-2012 [32]. The loading process was divided into two steps at the initial stage of the experiment, and it was load controlled at a rate of 2.5 kN/s. Thereafter, the displacement-controlled loading was adopted when 70% of the peak load was reached. The load was held for 10 min at each stage to measure the structural deformation and observe the crack development stably.

**Figure 6:**
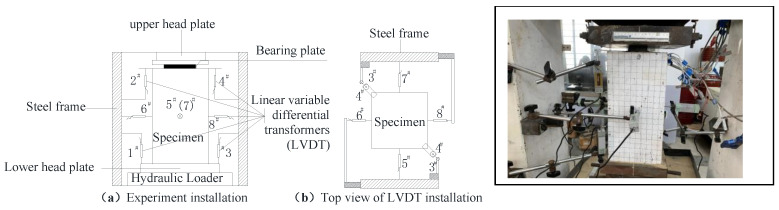
Test setup.

**Figure 7:**
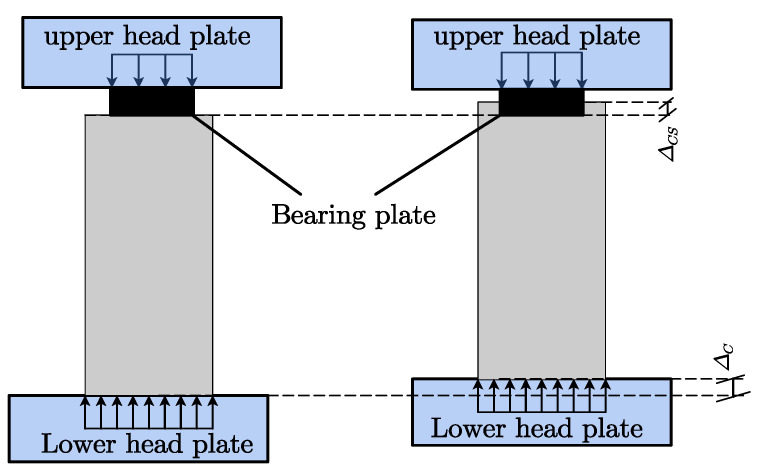
Compressive deformation of the UHPC specimen.

As illustrated in Figure 6, linear variable differential transformers (LVDT) were employed to measure the specimen’s deformation. The deformation of the specimen under local compressive load includes the depression of the bearing plate (that is, the indentation of the wedge-shaped body relative to its peripheral body) ${\;\Delta }_{cs}$, vertical deformation of the matrix in the specimen $\Delta_{cc}$, total vertical compression of the specimen under local load $\Delta_{c}$, and central expansion of the specimen under partial load $\Delta_{ce}$. Here, 2# and 4# LVDT were used to measure $\Delta_{ce}$, 1# and 3# were used to measure $\Delta_{c}$, and 5#–8# were used to measure $\Delta_{ce}$. $\Delta_{c}$ is the sum of the wedge-shaped body relative indentation deformation $\Delta_{cs}\;$and vertical compression deformation of the core body $\left(\Delta_{c}=\Delta_{cc}+\Delta_{cs}\right)$. Before the failure of the specimen, there was no obvious interface between the core matrix and the peripheral matrix. It can be approximately considered that the deformation of the two is coordinated, and the compression amount is the same, so the vertical compression value of the core matrix and the peripheral matrix of the specimen is $(\Delta_{cc}=\Delta_{c}-\Delta_{cs}$). These deformations were used to investigate the failure characteristics of the UHPC specimen, where $\Delta_{c}$ and $\Delta_{cs}$ deformations are illustrated in Figure 7. The final slip deformation value of the wedge-shaped body at the local compression zone $\Delta_{cs}^{\prime }$ was measured with vernier calipers after unloading.

## 3. Experimental Results and Discussions

### 3.1. Failure Characteristic

Prior to a load of 63% to 87% of the failure load, no phenomena were observed except the increase in deformation. When the cracking load was achieved, an initial crack appeared at the side surface of specimens, which corresponded to the middle of the bearing plate, accompanied by a stubble sound (slippage of steel fibers). The cracks occurred in the middle, where the tension stress reached the ultimate tensile strength of the UHPC.

During the loading process, the cracks propagated slowly, and the number of new cracks increased. Most of the cracks were spread in zone 300 mm or less from the loading end, and the majority were vertical with a small number of angled cracks. The broad and general zone where the largest bursting pressures occurred completely degraded as the cracks gradually developed with the loading. In all cases, bulging at the middle of the specimens was accrued. Before the failure, the loading rate slowed down, and the cracking sound was obvious, but the specimens were still in good integrity. The cracks developed rapidly when the peak load was achieved, with one or several dull cracking sounds (steel fiber pull out). Subsequently, the load suddenly dropped sharply at failure, and the specimen failed. The existing cracks expanded horizontally and extended vertically, and new cracks occurred near the existing ones. There was an obvious horizontal expansion deformation that started to increase significantly.

The load decreased sharply in the initial branch at the descending stage and then dropped gradually. When the load decreased to 70–80%, the UHPC specimens continued to bear load due to the efficient confinement of the stirrups. The concrete at the loading surface was slowly compacted until it lost its bearing capacity due to the loss of the stirrup’s confinement.

**Figure 8:**
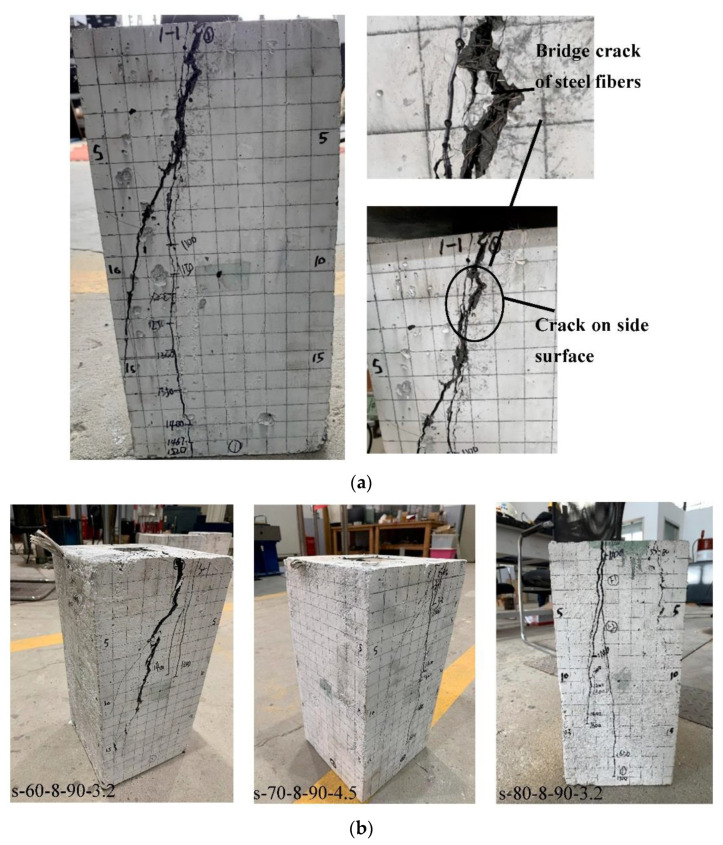
Failure characteristics of UHPC specimens. (**a**) Cracks propagation of the specimens; (**b**) specimens failure.

In all UHPC specimens, the concrete cover did not spall off due to the good confinement of steel fiber. Figure 8a shows that steel fiber bridge has one crack at the loading surface, while more than four cracks and depression of the bearing plat were observed. Figure 8b illustrates the failure characteristics of the specimens, and the bearing plate had been pressed into the sample by approximately 1 mm to 26 mm. After unloading, it was observed that the outer body at the loading end was separated from the core body. The failure characteristics of the specimens s-70-8-90-6.4 showed a significant ductility failure due to the high reinforcement ratio, while s-70-8-90-3.7 showed a less ductile failure compared with other specimens owing to the less sheet’s stirrups enhanced in the specimen, and s-60-8-90-3.2 bearing plate sunk inside the specimen after the load dropped to 80% of the peak load due to the relatively small width of the bearing plate. The rest of the specimens exhibited the same failure characteristics as explained above. After peeling off UHPC, it was found that there were obvious wedge-shaped body characteristics (pyramid shape) under the bearing plate. After failure, most of the specimens were relatively intact (in good integrity).

The experiment results demonstrate that the reinforcing significantly increases the bearing capacity. When the reinforcement ratio is 4.5%, the bearing capacity of s-70-8-90-4.5 increases by 23% compared with the s-70-8-90-3.7 reinforcement ratio of 3.7%. The influence of *A*_cor_/*A*_l_ and steel bar diameter is not obvious, where the bearing capacity only increased by 6% and 3%, while the *A*_cor_/*A*_l_ and the steel bar diameter increased from 70 to 110 and from 6 mm to 10 mm, respectively. In addition, increasing the bearing plate length from 60mm to 80 mm increases the bearing capacity to 19%.

The ratio of the cracking load to the peak load for the various specimens was from 63% to 87%. As shown in Table 4, the average ratio of the cracking and failure loads was approximately 72%. The results indicate that stirrups can significantly increase the specimen bearing capacity if it reinforces properly. However, on the other hand, it can also have a negative influence if the specimen was reinforced unproperly, as shown in s-70-8-90-6.4 and the literature [33], where the reinforcement failed to yield. However, the reinforcement was yielded in s-70-8-90-3.7 but still showed a negative influence on the bearing capacity of the specimen, due to less stirrup reinforcement being in the specimen, both designs should be avoided in structures.

### 3.2. Propagation of Cracks

**Figure 9:**
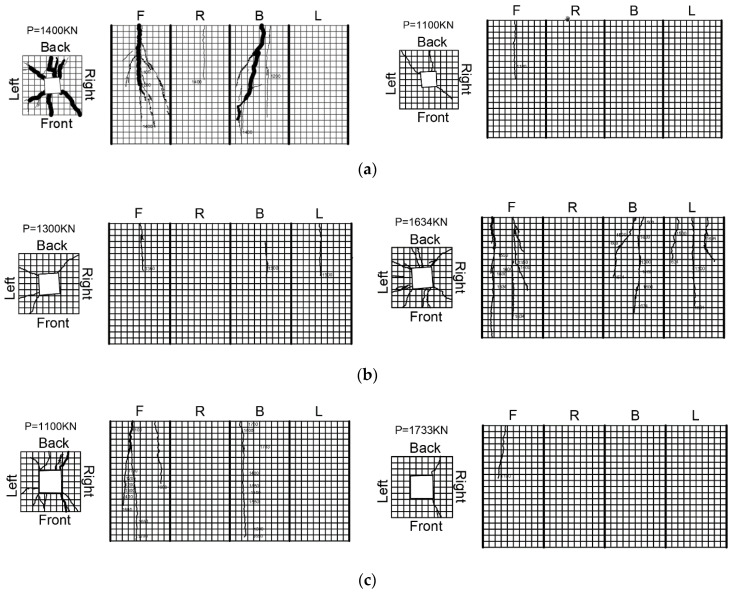
Crack propagation of UHPC specimens (**a**) s-60-8-90-3.2, (**b**) s-70-8-90-4.5, (**c**) s-80-8-90-3.2.

At the initial stage of the experiment, various specimens were loaded until 13–37% of the peak load *P***_u_**, and no cracks were observed. The specimen’s first crack was defined as the cracking load. The specimen’s cracks appeared in the height range from the central to the top surface, and the crack length was approximately equal to one or two widths of the bearing plate. The initial crack width was small, and the width along the crack development direction was uniform. The maximum initial crack width was 0.1 mm. One or two cracks appeared on the side center or near the edge of the bearing plate. Subsequently, as the load increased, the existing cracks slowly extended and widened, but there were few new cracks. The cracks propagation at the side surface indicated that the wedge cone under the bearing plate slips to some extent due to the confinement of steel fibers and reinforcement. The longitudinal cracks occurred and propagated at the side surface. Prior to peak load, new cracks developed rapidly and linked with the existing cracks. When the peak load was reached, new cracks appeared and developed rapidly. Among them, the lateral longitudinal cracks mainly occurred on the central axis or at the interface between the steel skeleton and the concrete; these show obvious “upper wide and lower narrow” cracks, and “wide outside and narrow inside” cracks appeared in the middle or edge or diagonal corner of the bearing plate. A wide crack appeared around the bearing plate at the loading end, but the specimen maintained its integrity. It was observed that the s-70-8-90-3.2, s-80-8-90-3.2, s-70-8-90-3.7, s-70-8-90-4.5, s-70-8-90-1.8, and s-70-8-90-5 UHPC specimens’ cracks extended to the specimen’s base. However, the rest of the UHPC specimens extended to the range of the middle to the base of the specimen. The crack’s characteristics are illustrated in Figure 9.

### 3.3. Wedge

**Figure 10:**
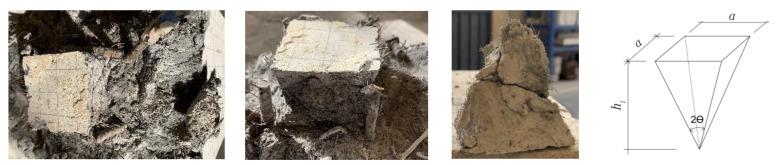
Wedge of UHPC specimens.

The failure characteristics, including the sinking of the bearing plate and vertical splitting cracks, confirmed the development of a wedge zone. The wedge was developed under the bearing plate. It was observed by peeling the UHPC. As shown in Figure 10, it can be considered a pyramid shape: the side length was measured as (*a*), and the height was (*h*_1_)_._ The tip angle is determined, and the results are shown in Table 5. The range of the tip angle was from 57° to 72°, and the average value of 2*θ* was 70°. A large wedge tip angle was observed compared to unreinforced specimens in the literature [27] due to the reinforcement effectively confining the development of the wedge.

### 3.4. Load Versus Deformation

**Figure 11:**
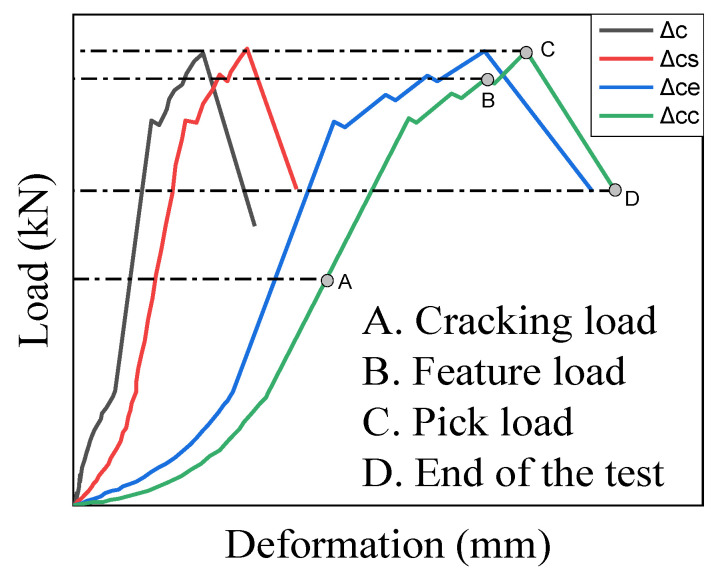
Load versus deformations model.

**Figure 12:**
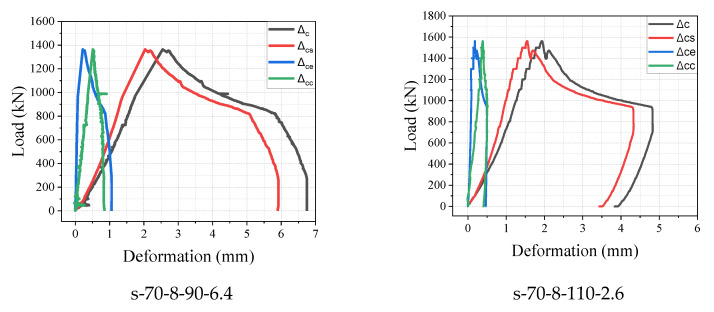
The local compressive load–deformation curve of UHPC specimens.

According to the relationship between local compression and deformation, deformations were divided into four basic three categories: $\left(\Delta_{c}\right)$, $\Delta_{cc}$, and $\Delta_{cs}$. All the deformations were analyzed during the whole loading process. At the beginning of the experiment, the load and deformations displayed a linear relationship. The concrete was compacted at the linear stage, cracks appeared gradually, and the reinforcement could not perform as anticipated. Figure 11 illustrates the relationship between deformation and load. Before the ultimate load, the deformations increased with the load, and after a few loading steps, a nonlinear relationship was obtained between the load and $\Delta_{c}$ until the feature load was achieved. In this paper, the term “feature load” is defined as the critical point at which the slope of the ascending branch dropped. Subsequently, numerous peaks could be observed in some specimens, the wedge was fully formed, and the confining reinforcement effect was constantly utilized. The peak load was achieved. After this, the load dropped rapidly, and the deformations decreased. The specimen continued to expand laterally at the third stage (reduction stage), utilizing the reinforcement’s remaining strength. Owing to the confinement of reinforcement and steel fibers, the wedge cone’s slippage was constant, proving the fact that $\Delta_{c}$ and $\Delta_{cs}$ continued to increase while the load dropped and $\Delta_{cc}$ decreased, the bearing capacity exhibited reduction owing to the crush of the concrete, while the remaining concrete continued to function. Figure 11 also illustrates that critical points A, B, C, and D can be defined as the cracking load, feature load, peak load, and end of the test, respectively. When the load was dropped to 70–80% of the peak load, the specimen continued to bear load due to the confinement of the stirrups, which provided considerable assurance of the structure’s safety. After that, the load dropped sharply. Due to the loss of the reinforcement confinement, a higher crushing failure occurred. Three types of relative deformations in the stage of wedge formation are shown in Table 6. Some of the specimen’s deformation curves are illustrated in Figure 12.

## 4. Finite Element Simulations

**Figure 13:**
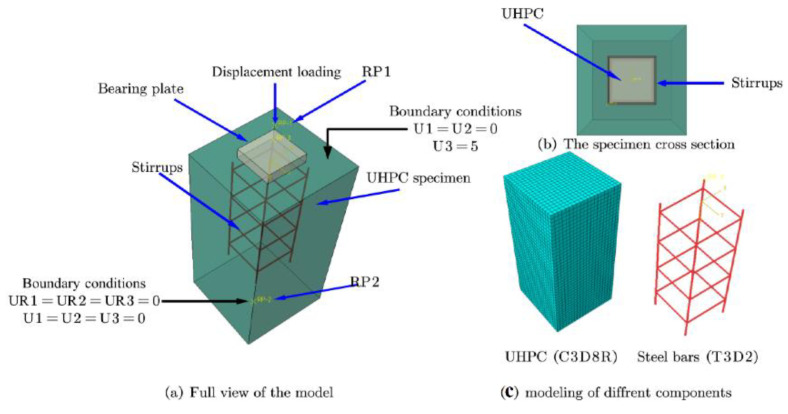
The numerical model for UHPC specimens.

Abaqus was used to perform a 3D nonlinear FEM analysis, and the model contains two major parts: the steel plate and the UHPC specimens. Therefore, FEM was used to simulate the UHPC specimens numerically. The finite-element model for UHPC specimens is shown in Figure 13.

### 4.1. Material properties

**Figure 14:**
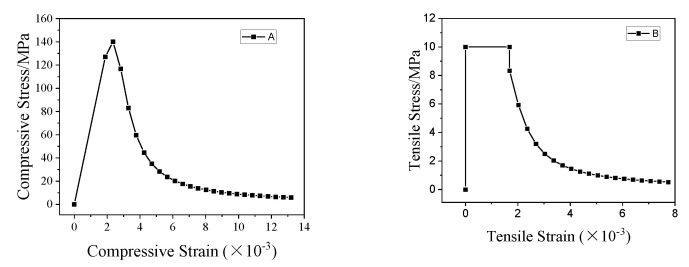
The compression and the tensile stress–strain curve of UHPC.

Due to a lack of relevant research data, this paper adopted the stress–strain constitutive model provided in the literature [34], and the tensile stress–strain curve of UHPC was developed. The main formula is shown in Equation (1) according to the uniaxial pressure stress–strain curve constitutive model given in the literature

(1) $$y=\{\begin{array}{c} ax+\left(5-4a\right)x^{4}+\left(3a-4\right)x^{5}\\ \frac{x}{b{\left(x-1\right)}^{2}+x} \end{array}$$

where: $x=\varepsilon /\varepsilon_{pr}$, $y=\sigma /\sigma_{pr\;}$, 1,1≤a≤1,4, 6≤b≤10, $\sigma_{pr}$—is the peak stress corresponding to the stress–strain curve of the constitutive model,$\;\varepsilon_{pr}$—is the strain at the peak stress corresponding to the stress–strain curve of the constitutive model. This paper takes a=1.18, b=6. The constitutive compression relationship of the UHPC specimens in this test is given. The compression, tensile peak stress, and corresponding strain of all specimens are shown in Table 7. The compression and the tensile stress–strain curve of all specimens are shown in Figure 14.

In addition, the plastic damage model provided in the finite element analysis software ABAQUS does not define the concrete constitutive relationship through stress–strain but defines the concrete constitutive relationship through stress-inelastic strain. Therefore, the above stress–strain constitutive relation needs to be obtained by the formula

(2) $$\varepsilon_{\mathrm{in}}=\varepsilon -\sigma /E_{0}$$

where:$\;\varepsilon_{\mathrm{in}}$—Inelastic strain, $E_{0}$ UHPC Elastic modulus.

Other material parameters in the damage plastic model in the finite element analysis software ABAQUS were: density ρ=2551 kg/m^3^, elastic modulus *E* = 58,200 MPa (the initial slope of the stress–strain curve), and Poisson’s ratio μ=0.19. To complete the yield surface and non-associated potential flow, the following data were provided in addition to the material properties:

- $f_{b0/}f_{c0}$ was modified to 1.16.
- $K_{c}$ was modified to 0.667
- μ was modified as 0.0005
- ∅ was modified with a default value of 0.1
- ψ/ was modified to 40°

where ψ is the dilation angle, ∅ is the eccentricity, f_b0_/f_c0_ is the ratio of biaxial pressure strength to uniaxial pressure ultimate strength, K_c_ is the constant stress ratio, defined as the ratio of the second stress invariant on the tensile meridian plane to the compression meridian plane, and μ is the viscosity parameter.

Unlike concrete, steel has a single stress–strain relationship that defines the material’s properties. The compressive and tensile regions were assumed to have the same stress–strain curve. As a result, this research examines numerical stability and computing efficiency while modifying material properties due to stress changes.

FEM analysis was performed by modeling the material’s behavior as an ideal plastic model. The steel’s elastic modulus (*E*_s_), Poisson’s ratio ($u_{s}$), and density were 2.0 × 10^5^ MPa, 0.3, and 7800 kg/m^3^, respectively. Other parameters are shown in Table 3.

Comparisons of the results obtained from FEM with the experimental results were described. Table 8 shows the analysis results together with the experiment regarding bearing capacity. The bearing capacity predicted by the FEM is close to the experiment results. It should be mentioned that the analysis performed in ABAQUS was in good agreement with the experimental results.

### 4.2. Type of Element and Mesh Sizes

Two-node linear truss elements (T3D2) were used to model reinforcements, and eight-node hexahedral elements (C3D8R) were used to model concrete. The embedding approach was used between the concrete and the reinforcement. It is important to note that ABAQUS has a variety of choices for modeling the interaction between reinforcement and concrete. The tension stiffening in the concrete model was used to incorporate the interaction between concrete and reinforcement after cracking, such as bond-slip and dowel action, and to apply boundary conditions comparable to the test setup. To ensure the model’s accuracy, the mesh size was adjusted to 10 mm for the UHPC, and the mesh size was adjusted to 5 mm for the reinforcement. The assembly assembled the reinforcement frame into UHPC using the EMBEDDED command so that reinforcement and UHPC could form a good interaction. The interactions with the steel plate and the upper part were applied through the command Tie.

### 4.3. Load Applications and Boundary Conditions

In order to apply the load applications and boundary conditions to match the anchorage zone behavior, two reference points were set at the specimen’s upper (RP1) and lower (RP2) parts. The steel plate and the lower part were coupled to the reference points RP1 and RP2, respectively. Based on the experiment, the reference point RP2 was flattened in three directions in the FEM to constrain the kinematic degrees of freedom and the torsion about the y-axis of the specimen. Furthermore, the UHPC specimen was loaded using displacement by applying load at the RP1, the displacement loading value of the RP1 reference point was set to 5 mm, and the analysis module STEP set the incremental step to 500.

### 4.4. FEM Results

All ten local compression finite element models were established according to the experiment’s geometric dimensions and related design parameters. Then all ten specimens were extracted in ABAQUS post-processing. The bearing capacity predicted by the simulations $P_{u,z}^{A}$ was compared with the test results $P_{u,z}^{T}$, and the comparison was presented in Table 8.

The error and mean error (M) were used to describe the overall model accuracy and the model’s average, overestimation, or underestimation. M (percentage) and error (percentage) are defined as follows:

$$\mathrm{Error}\left(\%\right)=\left|\frac{\mathrm{FEA}\;\mathrm{result}-\mathrm{Test}\;\mathrm{result}}{\mathrm{Test}\;\mathrm{result}}\right|\times 100$$

$$M\left(\%\right)=\frac{\mathrm{FEA}\;\mathrm{result}}{\mathrm{Test}\;\mathrm{result}}\times 100$$

To compare the finite element results with the experimental results, make $X=P_{u,G}^{A}/P_{u,G}^{T}$, then the average is $\bar{X}=1.03$, and the standard deviation is σ=0.052. It can be seen that the finite element results and the test results are in good agreement, indicating that the stress–strain relationship used in the finite element model material is more consistent with this test specimen.

In all situations, the model prediction of bearing capacity resulted in an error of less than 9%, as shown in Table 8. This demonstrates that the FEM’s predictions are similar to the experimental results. The simulated response of the test specimens was consistent with the experiment’s results. The simulated specimen confirmed that the indicated material parameters and constitutive model could detect the anchor zone’s behavior appropriately.

### 4.5. Analysis of Local Compression Failure Mechanism

**Figure 15:**
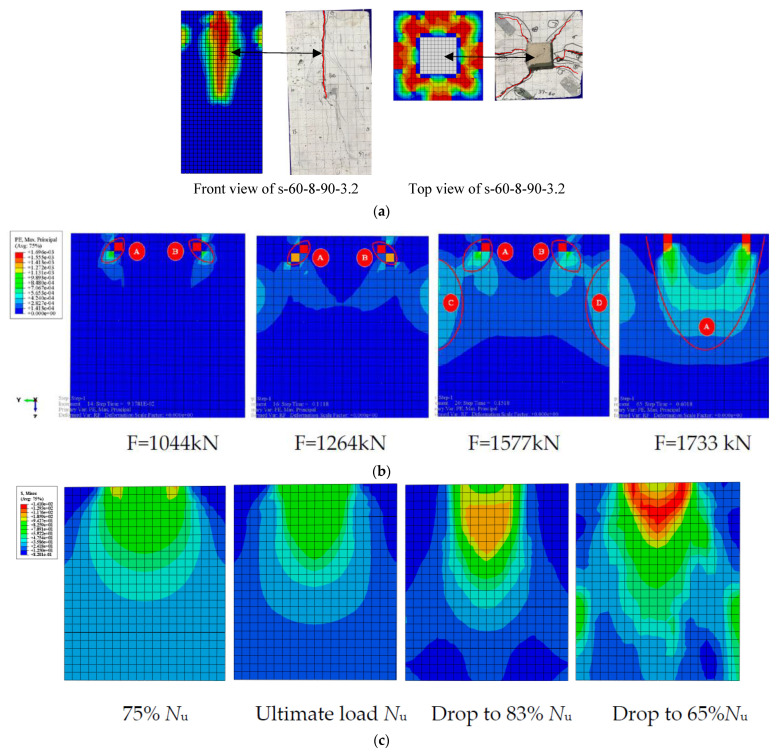

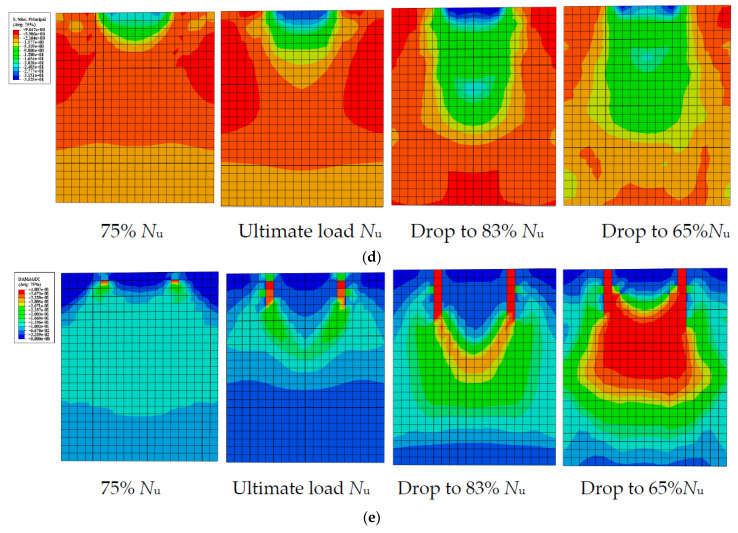
Results of numerical simulation. (**a**) Comparison of experimental and numerical failure modes; (**b**) Plastic maximum principal strain distribution of UHPC; (**c**) Mises stress distribution of UHPC; (**d**) Max. principal stress distribution of UHPC; (**e**) DAMAGEC distribution of UHPC.

This paper develops the FEM: PE, DAMAGEC, von Mises, and Max. Principal stress, respectively, for the local compression based on the constitutive model relationship of the UHPC gained from the test to understand better the failure mechanism of UHPC specimens under the influence of local loads. As illustrated in Figure 15a, the failure mode of UHPC specimens obtained from the numerical analysis are compared with the experiment results. The FE failure modes are consistent with the two deformed zones (side surface and top surface). The cracks and deformation position in the experiment are accorded with the numerical model. These results indicated that the numerical model could accurately predict failure modes and determine the failure mechanism of the UHPC specimen.

Figure 15b illustrated when the load reached 1044 kN the failure mechanism of the UHPC specimen’s parts A and B, the pattern develops downward in the syncline, and the maximum plastic principal strain gradually decreases, consistent with the diffusion of the local load inside the specimen. The maximum plastic principal strain initially appears outside the bearing plate at the initial stage of loading the specimen. The maximum plastic principal stresses of components A and B continue to grow diagonally downward as the load increases, as illustrated in Figure 15b when the load increases to 1264 kN. The bearing plate and the stresses in UHPC in sections A and B shaped an arch-like stress structure to support the local compression load, demonstrating that cracking happened when the ultimate tensile strain of the UHPC was obtained. The lateral tensile force initially occurred in the topper part of the specimen and gradually increased with loading. When the load reached 1577 kN, Figure 15b demonstrates that the maximum plastic principal strain is separated into four sections when the load grows to the maximum bearing capacity.

Additionally, as seen in Figure 15b, parts A and B have progressively connected after being disconnected from parts C and D. The primary reason is that the original arch structure created by A and B contains a shear slip plane, which led to the arch structure failing as the local stress increases and a wedge-shaped body was progressively formed. The wedge-shaped body in the specimen had fully developed, as seen in Figure 15b, when the load reached 1733 kN, and the component was damaged. This is demonstrated by the maximum plastic principal strain of part A being linked into parts.

In addition, further analyses were established, and the results of the numerical simulations are shown in Figure 15c–e. The Saint-Venant’s principle was confirmed by the von Mises stress distributions of the local compression UHPC specimen in Figure 15c. The highest stress was observed at the steel plate side from the loading end, where significant lateral deformation and damage were observed in the experiment. The development and downward slip of the wedge was also observable with the increase in stress. Due to the cover concrete’s extensive damage, the stress on the outside layer of the concrete disappeared after 80 to 65% of the peak load was obtained. The maximum principal stress distribution of the UHPC extension zone is shown in Figure 15d. The tensile stresses around the compressive zone and the compressive stresses under the steel plate were the regions that consisted primarily of the stress distribution zone. At the initial loading stage, the tensile stress zone was observed at the lower part of the steel plate and the specimen side surface. The cracks occurred when the tensile stress was more significant than the tensile strength of the UHPC.

Each part of the tensile zone was gradually joined with the load increase, which is similar to the propagation of the cracks in the experiment. A pyramid shape was gradually formed in the compressive zone under the bearing plate (wedge). When the peak load was reached, the wedge was moved downward, and due to the tensile stress around the edge of the wedge and splitting of the wedge, the compressive zone continued to expand downward. It was easy to determine from the analysis of the specimens’ during the loading process that the split mechanism of the specimen under local compression conforms to the wedge split theory. The wedge development process and action mechanism were shown in the DAMAGEC distribution of UHPC. The formation and downward slip of the wedge was obvious with the increase in stress, as illustrated in Figure 15e. The whole failure process of the UHPC specimen can be described as follows: (i) damage initially accrued at the beginning of the outer side of the steel plate, (ii) the damage gradually devolved downward, (iii) wedge has developed and gradually sunken inside the specimen, and (iv) failure of the UHPC specimen.

### 4.6. Investigation of Material Parameters

**Figure 16:**
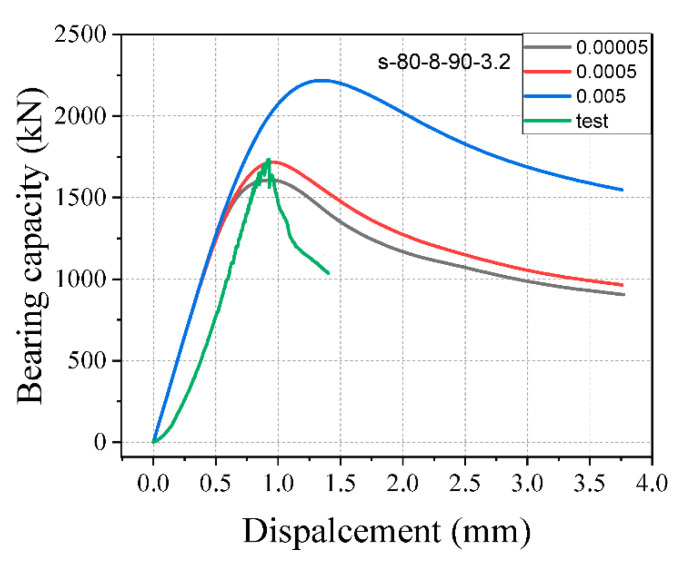
Effect of different viscosity parameters.

To discuss the parameters of the CPD model, specimen s-80-8-90-3.2 was chosen as a control specimen. The viscosity parameter had different values in each of the cases studied. μ are 0.00005, 0.0005, and 0.005. When the viscosity parameter was set as 0 (ABAQUS’s default value), the viscosity parameter’s value was proportional to the time increment step. It is recommended that a value of roughly 15% of the time increment step be assigned to improve the solution [35]. The time increment step was not fixed and was set automatically. Viscosity parameters set to 0.00005, 0.0005, and 0.005 in terms of bearing capacity and displacement were discussed. The results revealed that giving the model small values for the viscosity parameter can underestimate the bearing capacity of the specimen, and assigning the big values can overestimate the bearing capacity of the specimen. Therefore, assigning 0.0005 viscosity parameters can improve the accuracy of the model. Results of various viscosity parameters are shown in Figure 16.

The computational time for the analyses using viscosity parameters of 0.00005, 0.0005, and 0.005 was 9 min, 7 min, and 6 min, respectively. Increasing the viscosity parameters reduces the computing time by allowing for a more considerable stable time increment, but it can influence the model’s accuracy. The results revealed that the viscosity parameter value should be chosen carefully when utilizing the CDP model in practical computations. The analysis results indicated that setting a suitable viscosity parameter to the model can improve the model’s accuracy. This paper used the static analysis with a 0.0005 viscosity parameter.

The effect of mesh size on the results was investigated. To investigate the level of mesh dependency of the model, three various mesh sizes (10 mm, 12 mm, and 14 mm) were used in the specimen analysis. The analyses were presented with 10 mm, 12 mm, and 14 mm mesh sizes. Twenty elements were considered across the specimen depth using elements with a mesh size of 10 mm. In contrast, 17 and 14 elements were constructed using mesh sizes of 12 mm and 14 mm, respectively.

**Figure 17:**
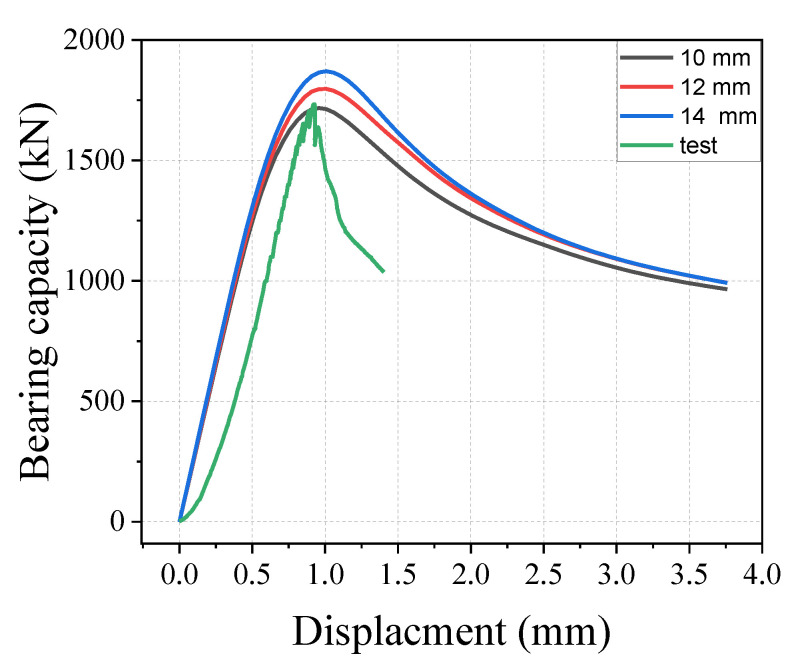
Effect of the different mesh sizes.

Figure 17 shows the results of the bearing capacity–displacement curve, indicating that mesh sizes of 10 mm were similar and in good alignment with the experimental test results. While mesh sizes of 12 mm and 14 mm overestimated the bearing capacity of the specimen, indicating that the small mesh can achieve higher accuracy calculation for the model. The models with mesh sizes of 10 mm, 12 mm, and 14 mm took 7 min, 3 min, and 2 min for computation, respectively. The computing time can be significantly reduced by increasing the mesh size. In the following simulations, a mesh size of 10 mm was chosen based on the bearing capacity of the model.

### 4.7. Parametric analysis

The consideration of the reinforcement ratio in the local compression test of the UHPC with reinforcement is significant. This paper establishes a local compression finite element model based on the relevant constitutive relationship of material in Equation (1). It uses the reinforcement spacing as a variable to apply the parametric analysis, specimen s-80-8-90-3.2 was set as the basis for the parametric analysis.

In this study, a nonreinforced specimen and five specimens with different reinforcement spacing were investigated to explore the contribution of the reinforcement and the reinforcement spacing to the bearing capacity of the specimens.

**Figure 18:**
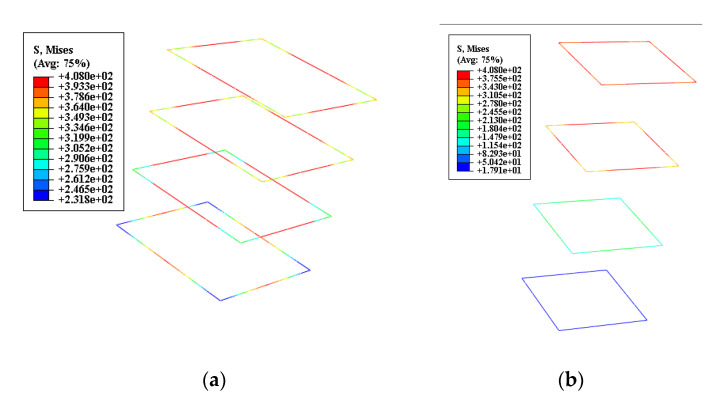
Different stirrups spacing stress contour. (**a**) 40 mm; (**b**) 80 mm.

The reinforcement ratio can be calculated by $p_{v}=nA_{s}/\left(l_{Cor}s\right)$. The corresponding $P_{v}$ reinforcement ratios were 0% to 5%, where$\;l_{Cor}\;,n,\;\;\mathrm{and}\;A_{s}$ were taken as 90 mm 4 and 50.24 mm^2^, respectively. To maximize the expansion of the number of specimens, the space between reinforcement bars was taken as 40 mm, 50 mm, 60 mm, 70 mm, and 80 mm, respectively. The steel bar diameter was 8 mm. Figure 18 shows different stirrups spacing stress contours. The parameters of the specimen and the corresponding local compressive bearing capacity are shown in Table 9, where$\;P_{u,H}^{W}$ is the local compressive bearing capacity, and $P_{u,J}^{W}$ is the contribution of reinforcement in local compressive bearing capacity.

**Figure 19:**
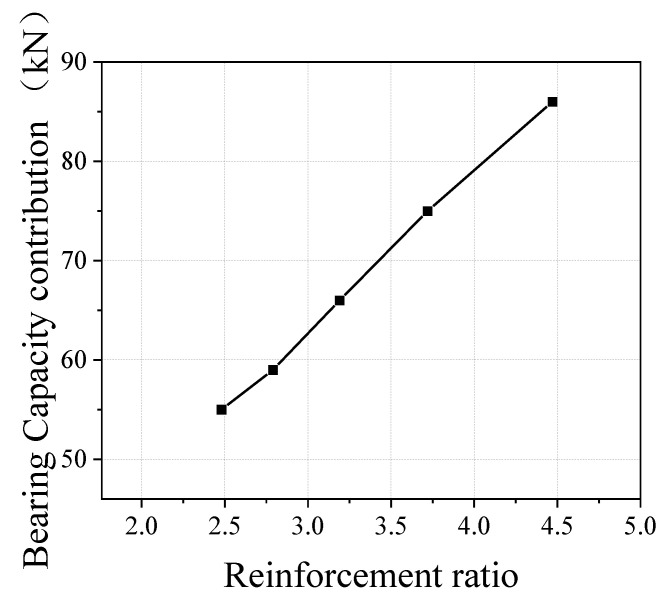
Fitting of reinforcement ratio parameters.

In this paper, the reinforcement ratio was taken as the abscissa, the local compressive bearing capacity contributed by the reinforcement was used as the ordinate, and the relationship between the two was analyzed, as shown in Figure 19.

According to the linear fitting of the reinforcement ratio parameter, the linear regression correlation coefficient is 0.98, which is close to 1. The reinforcement ratio *p*_v_ is highly correlated with the reinforcement contribution in terms of the local compressive bearing capacity of the UHPC specimen. UHPC also has a linear relationship between the reinforcement ratio (reinforcement spacing) and the local compressive bearing capacity contributed by reinforcement.

## 5. Bearing Capacity Calculation Method

Many scholars have worked hard to establish the bearing capacity, and several formulae have been suggested. The purpose is to accurately calculate the local bearing capacity and recommend a sufficient method to design specimens under local compression. The test results showed that when the peak load was attained, the actual stress of the stirrups in some of the UHPC specimens did not perform well, i.e., some of the stirrups did not yield when the peak load was achieved.

In this instance, it is important to find a model that can accurately predict the bearing capacity of UHPC specimens. This part presents a model to predict the UHPC specimen bearing capacity. The calculated values using the model have shown high accuracy in predicting the bearing capacity for 10 UHPC specimens, the model can be set as a reference to give an efficient design of UHPC specimens under local compression.

### 5.1. Development of Model for UHPC Confined with Stirrups

An equation was introduced by Zheng et al. [29] to predict the local bearing capacity of plain RPC specimens.

(3) $$N_{u,c}=\beta_{1\mathrm{RS}}f_{c}A_{ln}$$

where $\beta_{1\mathrm{RS}}$ is the influence coefficient of local compressive strength of UHPC mixed with steel fiber considering the curing condition $\left(\beta_{1\mathrm{RS}}=0.7\beta_{l}+0.1\right)$, $\beta_{l}$ is the square roots of the local area aspect ratio ($\beta_{l}=\sqrt{A_{b}/A_{l}}$), $f_{c}$ is the compressive strength of concrete, and $A_{ln}$ is the net area of the bearing plate.

An equation was published by the “Code for the design of concrete structures” (GB50010-2010 [36] for calculating the local compressive bearing capacity contributed by the reinforcement and is given as:

(4) $$N_{u,s}=2\alpha \beta_{cor}p_{v}f_{y}A_{ln}$$

where: α is the reduction coefficient of reinforcement for concrete restraint, which is taken as 0.85,$\;p_{v}$ is the reinforcement ratio,$\;\beta_{cor}$ is the square root of the area confined by stirrups $\beta_{cor}=\sqrt{A_{cor}/A_{l}}$,$\;f_{y}\;$is the yield strength, and$\;A_{ln}\;$is the net area of the local compression of concrete.

Equation (3) is for calculating the local bearing capacity of plain RPC specimens, and Equation (4) is for calculating the local compressive bearing capacity contributed by the reinforcement. A combined formula was introduced to predict the bearing capacity of reinforced UHPC without duct. Considering the actual stress generated by the stirrups, the following model is presented below:

(5) $$N_{u}=A_{ln}\left(2\alpha \beta_{cor}p_{v}f_{y}+\beta_{1\mathrm{RS}}f_{c}\right)$$

This model is useful for calculating the bearing capacity of UHPC reinforced with stirrups, the specimen’s design should consider structural codes.

### 5.2. Comparison between Experimental Results and Prediction Model

Regardless of the specimens with or without duct, the stress mechanism of the steel fiber UHPC under the action of local compression is consistent. Therefore, to ensure the formula’s accuracy, the calculation formula of UHPC local compressive bearing capacity is unified to make it more adaptable and convenient.

According to Equation (3), the contribution of UHPC to bearing capacity under local compression$\;N_{u,c}$ was calculated, and the contribution of reinforcement to bearing capacity under local compression $N_{u,s}$ was calculated by Equation (4), and the calculated value of the local compression bearing capacity of the UHPC of the specimens $N_{u}$ can be obtained by Equation (5). Calculated results are shown in Table 10.

Further, to compare the calculated results with the experimental results, the standard deviation (SD), the average values (AV), and coefficient of variation (CV) were employed to evaluate the accuracy of the model.

Table 10 compares the calculated results and the experiment results. The results indicated that the model could accurately predict the bearing capacity of reinforced UHPC, the SD, AV, and CV were calculated using the introduced model for predicting the local bearing capacity, not considering s-70-8-90-6.4 and s-70-8-90-3.7 are 0.04,1.04, and 3.5%, respectively, but considering s-70-8-90-6.4 and s-70-8-90-3.7 are 0.11,1.09, and 10.2%. This Indicates that the formula used to calculate the bearing capacity is more consistent with these test specimens, and it also proves that the curing conditions influence the local compressive bearing capacity of the UHPC, and the reinforcement content has an obvious influence on the local compressive bearing capacity of UHPC.

The calculated bearing capacity of s-70-8-90-3.7 and s-70-8-90-6.4 were superior to the test results. It indicated that the reinforcement did not play a role in improving the bearing capacity. However, it had a negative influence on weakening the specimen. The specimen did not exhibit its full-time bearing capacity, so it is not recommended to use this type of design specimen. i.e., over-reinforcing or enhancing less than four stirrups is not recommended during the design of the UHPC anchorage zone specimen. The rest of the specimens agree with the experimental results compared to the calculated values without considering s-70-8-90-3.7 and s-70-8-90-6.4, and all specimen errors were less than 7.5%, as shown in Table 10. It again demonstrates that the calculated values are similar to the experimental results.

## 6. Conclusions

Ten UHPC specimens were tested in this study to analyze the influence of local compression reinforced with stirrups on the mechanical characteristics of UHPC. The following conclusions were taken from the findings of this study:

1. In order to simulate the reinforced UHPC specimens being subjected to local compression, a numerical model was created. In terms of the failure mode and local bearing capacity, the numerical results were in good agreement with the experiment results. The reinforced UHPC specimens displayed a ductile failure behavior. When the UHPC specimens were subjected to a load close to the peak load, a wedge-formed under the bearing plate and started to slide downward. From the simulation, the failure characteristics of UHPC conform to the wedge split theory.
2. The experiment results demonstrate that the reinforcing significantly increases the bearing capacity. When the reinforcement ratio is 4.5%, the bearing capacity of s-70-8-90-4.5 increases by 23% compared with the s-70-8-90-3.7 reinforcement ratio of 3.7%. The influence of *A*_cor_/*A*_l_ and steel bar diameter is not obvious, where the bearing capacity only increases by 6% and 3%, when the *A*_cor_/*A*_l_ and the steel bar diameter increased from 70 to 110 and from 6 mm to 10 mm, respectively. In addition, increasing the bearing plate length from 60 mm to 80 mm increases the bearing capacity to 19%.
3. When the reinforcement ratio of the stirrups is $\rho_{v}$ > 6.4%, the stirrups cannot attain the yield strength at the peak load and can continue to confine the core concrete over the peak load. After the ultimate load is reached, the stirrups are susceptible to yield. The stirrups within UHPC under local compression exhibited a similar confinement characteristic to the ordinary concrete. Stirrups significantly increase the ductility and safety reserve of UHPC specimens, allowing the specimens to preserve integrity throughout the complete loading process.
4. A model was proposed to calculate the bearing capacity of reinforced UHPC specimens, and the model accurately predicts the local bearing capacity for the UHPC specimens confined with stirrups. Each design of reinforced UHPC specimens should put into consideration the design codes.
5. This paper provides a solid foundation for designing the UHPC prestressed members, ensuring the safety and serviceability of structures. Moreover, there is no significant research for predicting the UHPC specimens confined with fiber reinforced polymer bar, enhancing glass fiber, and UHPC specimens under various curing conditions.

## Figures and Tables

**Table 1:** Proportioning of the UHPC mix.

Cement	Silica Fume	Quartz Sand	Water to Cementitious Material	Super Plasticizer	Steel Fiber	Ground Slag to Cement
1	0.3	1.2	0.16	4	2.5%	0.15

**Table 2:** Mechanical properties of steel bars.

Diameter	*f*_y_ (MPa)	*f*_u_ (MPa)	*E*_s_ (MPa)	*ε*_sv_ (με)
6	480	640	200,000	2400
8	408	629	196,531	2076
10	431	632	192,582	2238

Note: *f*_y_ is the yield strength; *f*_u_ is the ultimate strength; *E*_s_ is the elastic modulus; *ε*_sv_ is the yield strain.

**Table 3:** Mechanical properties of UHPC specimens.

Parameters	No.	*A* (mm)	*A*_b_/*A*_l_	*d* (mm)	*l*_cor_ (mm)	*A*_cor_/*A*_l_	*p*_v_ (%)
Bearing plate length	s-60-8-90-3.2	60	11.1	8	90	2.3	3.2
	s-70-8-90-3.2	70	8.2	8	90	1.7	3.2
	s-80-8-90-3.2	80	6.3	8	90	1.3	3.2
Reinforcement configuration	s-70-8-90-3.7	70	8.2	8	90	1.7	3.7
	s-70-8-90-6.4	70	8.2	8	90	1.7	6.4
	s-70-8-90-4.5	70	8.2	8	90	1.7	4.5
The length of the inner edge of the reinforcement sheet steel bar	s-70-8-70-4.1	70	8.2	8	70	1	4.1
	s-70-8-110-2.6	70	8.2	8	110	2.5	2.6
Steel bar diameters	s-70-6-90-1.8	70	8.2	6	90	1.7	1.8
	s-70-10-90-5	70	8.2	10	90	1.7	5

Note: in the nomenclature of the specimens, the letters and numbers respectively represent bearing plate length, steel bar diameter, the inner edge of the reinforcement sheet steel bar, and the reinforcement ratio.

**Table 4:** Failure loads and cracking.

No.	*P*_cr_ (kN)	*P*_u_ (kN)
s-60-8-90-3.2	1100	1400
s-70-8-90-3.2	1000	1552
s-80-8-90-3.2	1100	1733
s-70-8-90-3.7	800	1226
s-70-8-90-6.4	1000	1387
s-70-8-90-4.5	1300	1634
s-70-8-70-4.1	1050	1464
s-70-8-110-2.6	1100	1556
s-70-6-90-1.8	1100	1519
s-70-10-90-5	1100	1576

**Table 5:** Wedge angle.

No.	2θ
s-60-8-90-3.2	69°
s-70-8-90-3.2	66°
s-80-8-90-3.2	72°
s-70-8-90-3.7	71°
s-70-8-90-6.4	72°
s-70-8-90-4.5	69°
s-70-8-70-4.1	60°
s-70-8-110-2.6	65°
s-70-6-90-1.8	57°
s-70-10-90-5	68°

**Table 6:** Relative deformation in the stage of the wedge formation.

No.	$\Delta_{c}/P\;\;(\mu m/\mathrm{kN})$	$\Delta_{cc}/P\;(\mu m/\mathrm{kN})$	$\Delta_{cs}/P\;(\mu m/\mathrm{kN})$	$\Delta_{ce}/P\;(\mu m/\mathrm{kN})$	$\Delta_{cs}^{\prime }\;(\mathrm{mm})$
s-60-8-90-3.2	2.953	0.859	2.103	0.100	1.82
s-70-8-90-3.2	2.361	0.793	1.568	0.129	3.56
s-80-8-90-3.2	2.265	0.823	1.474	0.243	1.8
s-70-8-90-3.7	2.829	0.385	2.444	0.265	2.35
s-70-8-90-6.4	2.473	0.611	1.963	0.184	2.82
s-70-8-90-4.5	1.638	0.681	1.957	0.088	2.35
s-70-8-70-4.1	1.371	0.450	0.923	0.067	1.16
s-70-8-110-2.6	1.931	0.386	1.545	0.105	2.32
s-70-6-90-1.8	2.293	0.679	1.621	0.130	0.86
s-70-10-90-5	2.533	0.836	1.706	0.245	1.88

**Table 7:** UHPC Specimen compression and tensile peak stress and strain.

$\sigma_{pr}^{c}/MPA$	$\varepsilon_{pr}^{c}$	$\sigma_{pr}^{t}/MPA$	$\varepsilon_{pr}^{t}$
140	0.002357	10	0.000168

**Table 8:** Comparison of experimental results with numerical calculation results.

No.	$P_{u,G}^{T}/\mathrm{kN}$	$P_{u,G}^{A}/\mathrm{kN}$	$P_{u,G}^{T}/P_{u,G}^{A}$
s-60-8-90-3.2	1733.2	1717.582	1.01
s-70-8-90-3.2	1552.4	1579.101	0.98
s-80-8-90-3.2	1400.1	1381.452	1.01
s-70-8-90-3.7	1464.2	1385.1	1.06
s-70-8-90-6.4	1562.4	1428.426	1.09
s-70-8-90-4.5	1226	1288.234	0.95
s-70-8-70-4.1	1387	1276.024	1.09
s-70-8-110-2.6	1634.3	1618.765	1.01
s-70-6-90-1.8	1519.2	1583.772	0.96
s-70-10-90-5	1576.7	1432.831	1.1
Mean			1.03
SD			0.052

**Table 9:** Specimen parameters and partial pressure bearing capacity.

s	$P_{v}$	$P_{u,H}^{W}$	$P_{u,J}^{W}$
0	0	1663	0
40	4.47	1747	86
50	3.72	1738	75
60	3.19	1727	66
70	2.79	1718	59
80	2.48	1711	55

**Table 10:** Comparison of experimental results with calculation results.

No.	$N_{u,s}$	$N_{u,c}$	$N_{u}$	$N_{u}^{T}\;$	Error%
s-60-8-90-3.2	120	1225	1366	1400	4.08%
s-70-8-90-3.2	140	1442	1606	1552	4.36%
s-80-8-90-3.2	160	1658	1846	1733	4.64%
s-70-8-90-3.7	162	1442	1632	1226	25.46%
s-70-8-90-6.4	280	1442	1771	1387	19.44%
s-70-8-90-4.5	197	1442	1673	1634	0.28%
s-70-8-70-4.1	139	1442	1606	1464	7.42%
s-70-8-110-2.6	139	1442	1605	1556	1.57%
s-70-6-90-1.8	79	1442	1535	1519	0.11%
s-70-10-90-5	218	1442	1699	1576	5.09%

## Data Availability

Not applicated.
